# Keratoconic eyes with stable corneal tomography could benefit more from custom intraocular lens design than normal eyes

**DOI:** 10.1038/s41598-019-39904-w

**Published:** 2019-03-05

**Authors:** Simon Schröder, Timo Eppig, Weidi Liu, Jens Schrecker, Achim Langenbucher

**Affiliations:** 1 Saarland University, Institute of Experimental Ophthalmology, Kirrberger Str. 100, Bldg. 22, D-66424 Homburg/Saar, Germany; 2 University of Rochester, Institute of Optics, 275 Hutchison Road, Rochester, NY 1427-0186 USA; 3 Rudolf-Virchow-Klinikum Glauchau, Department of Ophthalmology, Virchowstr. 18, D-08371 Glauchau, Germany; 4 Rice University, 301 Space Science, 6100 Main, St Houston, TX 77005 USA

## Abstract

We investigated whether eyes with keratoconic corneal tomography pattern could benefit more from aberration correction with custom intraocular lenses (IOLs) than normal cataractous eyes despite the effect of misalignment on the correction of aberrations. Custom IOLs (cIOLs) were calculated for twelve normal and twelve keratoconic eyes using personalized numerical ray tracing models. The Stiles-Crawford weighted root-mean-square spot-size (wRMS) at the virtual fovea was evaluated for cIOLs and aberration-neutral IOLs (nIOLs) in a simulated clinical study with 500 virtual IOL implantations per eye and per IOL. IOL misalignment (decentration, tilt, rotation) and pupillary ectopia (4.5 mm iris aperture) were varied upon each virtual implantation. The nIOLs achieved average wRMS of 16.4 ± 4.3 μm for normal, and 92.7 ± 34.4 μm for keratoconic eyes (mean ± standard deviation). The cIOLs reduced the average wRMS to 10.3 ± 5.8 μm for normal, and 28.5 ± 18.6 μm for keratoconic eyes. The cIOLs produced smaller wRMS than nIOLs in most virtual implantations (86.7% for normal and 99.4% for keratoconic eyes). IOL misalignment resulted in larger wRMS variations in the keratoconus group than in the normal group. Custom freeform IOL-optics-design may become a promising option for the correction of advanced aberrations in eyes with non-progressive keratoconic corneal tomography pattern.

## Introduction

Cataract is characterized by the natural human lens becoming opaque leading to significant loss of vision^1^. It is treated by surgical removal of the lens and subsequent replacement with an artificial intraocular lens (IOL). Numerical ray tracing can be used to choose the IOL’s refractive power^2–4^ and cylinder^5^ accurately to achieve low postoperative refractive errors, and to estimate the appropriate asphericity of the IOL^6^ in order to correct higher order aberrations of the eye. An IOL with customized asphericity can result in improved postoperative contrast sensitivity for normal cataractous eyes^7^.

The coma aberration coefficients of postoperative aberrometry can be reliably predicted in addition to spherical aberration, and spherical and cylindrical refractive errors^8^. Improvements of postoperative vision might be possible with custom IOLs that correct higher order aberrations in addition to spherical aberration and refractive error (sphere, cylinder). Different concepts have been developed to customize the anterior or posterior optic design of IOLs^9–12^. The IOL’s posterior surface and edge design is important to prevent posterior capsular opacification^13^. Customization of the anterior IOL optic might be preferable.

Keratoconus is a progressive, asymmetrical corneal degeneration resulting in corneal thinning and a conical protrusion of the cornea^14^. The protrusion results in myopia, (irregular) astigmatism, and further aberrations that limit the visual performance and cannot fully be corrected by spectacles. Corneal cross-linking is often used to prevent further progression^15^. The use of custom IOLs for the correction of advanced corneal refractive errors associated with keratoconus has been suggested^11^.

To use corneal measurement in numerical ray tracing, corneal elevation is often represented by a number of Zernike polynomials^4,10^. However, the Zernike representations of many keratoconic corneas with a reasonable number of polynomials fail to represent all corneal aberrations which affect visual function^16,17^. Alternative ways to represent the cornea have been suggested to facilitate the ray tracing analysis of asymmetric corneas^14,18–20^.

IOL misalignment reduces the image quality with custom IOLs significantly^21–23^. With IOL decentration <0.3 mm, the image quality with custom IOLs is expected to be superior to the image quality with standard IOLs. However, the combination of IOL tilt and decentration can result in different outcomes^24^. Monte Carlo analysis has been used to study the impact of combined IOL tilt and decentration and the influence of uncertainties on image quality metrics with (custom) IOLs^10,25,26^.

In this manuscript, we investigate the potential benefit of custom IOLs for normal cataractous eyes and keratoconic eyes for monochromatic light in the presence of IOL misalignment to determine if eyes with nonprogressive keratoconic corneal tomography pattern could benefit more from aberration correction with custom IOLs than normal cataractous eyes.

## Results

### Patient Data

Measurements from twelve eyes (twelve patients) without history of ocular surgery (six right, six left eyes) were included in each of the two groups (Table 1): patients with normal cataractous eyes (normal group) and patients with keratoconic eyes (keratoconus group). To illustrate the IOL optimization and the effect of IOL misalignment on the Stiles-Crawford weighted root-mean-square spot-size (wRMS), the results of two left eyes are shown in detail: One (NP) belongs to the normal group the other (KP) to the keratoconus group. Both required an aberration-neutral IOL (nIOL) with 22.5D to achieve a minimal wRMS.

**Table 1:** Biometric data from normal cataractous eyes and keratoconic eyes (mean ± standard deviation, range).

Group	Eye	*K*_*f*_ (*D*)	*K*_*s*_ (*D*)	ACD (mm)	AL (mm)	TKC
normal	right	43.05 ± 1.04 (41.2…43.8)	43.55 ± 0.96 (41.8…44.3)	3.29 ± 0.31 (2.69…3.55)	23.79 ± 0.74 (22.67…24.63)	0
	left	43.22 ± 1.84 (41.6…45.8)	43.85 ± 2.05 (41.8…46.4)	3.47 ± 0.43 (2.84…3.80)	23.32 ± 0.35 (22.85…23.81)	
keratoconus	right	44.60 ± 1.70 (43.1…47.2)	47.65 ± 2.36 (44.1…50.1)	3.47 ± 0.28 (3.20…3.91)	22.91 ± 0.84 (22.91…25.4)	2.17 ± 0.75 (1…3)
	left	42.10 ± 1.95 (39.2…44.7)	46.67 ± 2.62 (43.5…49.4)	3.51 ± 0.52 (2.93…4.46)	23.93 ± 1.04 (22.75…25.24)	2.42 ± 0.92 (1…3.5)
NP	left	41.9	42.2	2.84	23.35	0
KP	left	41.1	43.6	2.93	23.23	1

K_f_, K_s_: keratometric readings, ACD: anterior chamber depth, AL: axial length, TKC: topographic keratoconus classifier, NP: normal patient, KP: keratoconus patient.

### Corneal Representation

To use the corneal tomography for the ray tracing based calculation of the IOL and modeling of the pseudophakic optics, the corneal measurements (anterior and posterior corneal surface) were represented with a mathematical surface model consisting of 11 Zernike polynomials (*j* ≤ 11^27^) plus fourth order basis-splines^28^. The surface model fitted the corneal elevation data with a residual error (weighted root-mean-square, *χ*) of <0.32 µm for the anterior cornea and <0.36 µm for the posterior cornea of normal and keratoconic eyes. An approximation with 28 Zernike polynomials (*j* ≤ 28^27^) was insufficient to achieve $\chi \le \sqrt{\sum _{i}1/{\sum }_{i}{s}_{i}^{-2}}$ for eleven out of twelve anterior corneal surfaces of the keratoconic eyes, where *s*_*i*_ refers to the precision of the measurement with Pentacam HR for normal eyes^29^ at measured data point *i* and the sums are over all valid data points. More than 80% of the data points of the anterior corneal topography within 10 mm diameter were considered valid for each eye.

### IOL Optimization

**Figure 1:**
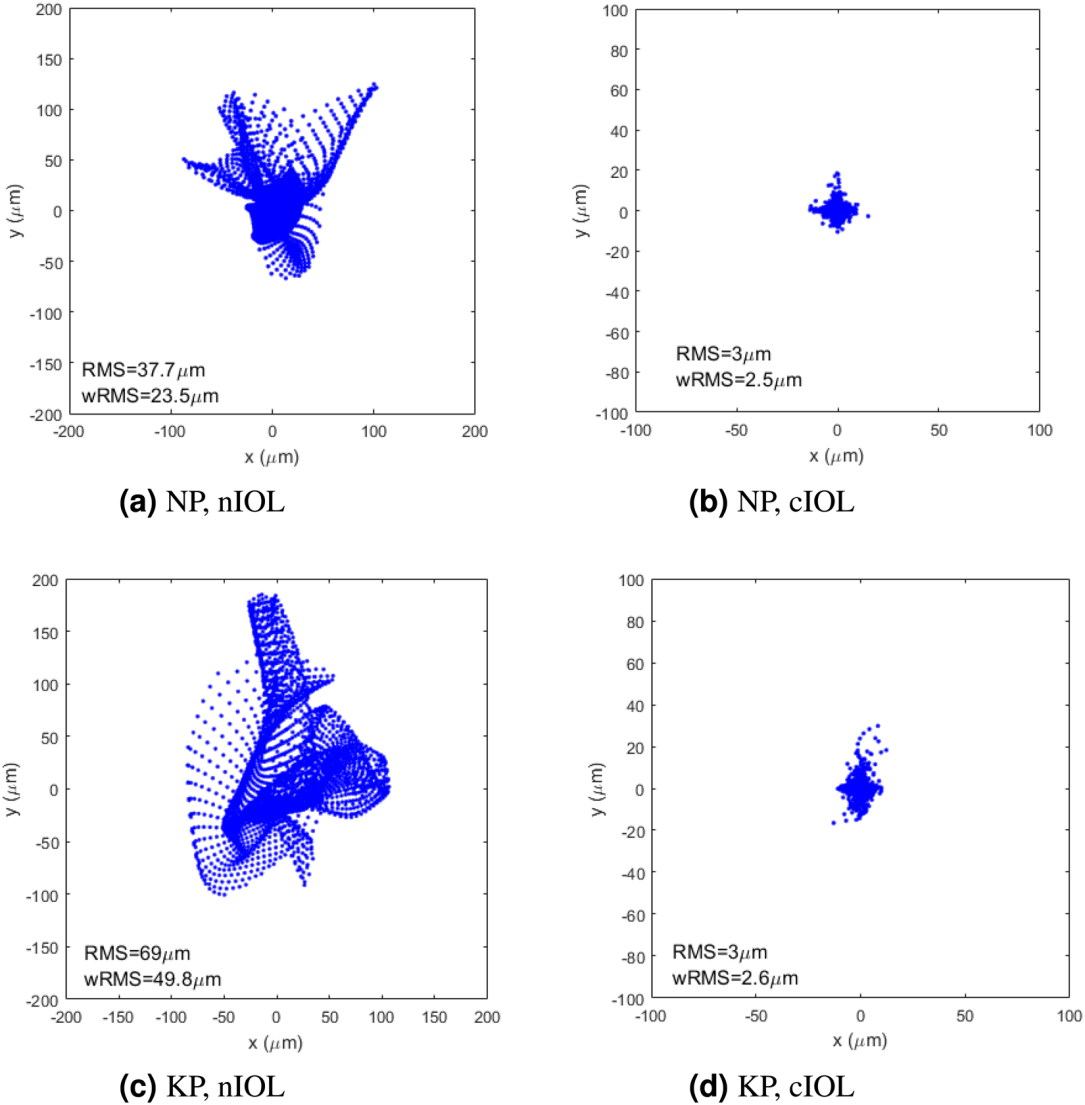
The spot diagrams achieved with the centered intraocular lens (IOL) for normal eye NP (top) and keratoconic eye KP (bottom) with an aberration-neutral IOL (nIOL, left) and a custom IOL (cIOL, right). The inlay displays the root-mean-square spot-size (RMS) and the weighted root-mean-square spot-size (wRMS). For better visibility, the spot diagrams of the cIOLs are zoomed in by a factor of two compared to those with the nIOLs.

**Figure 2:**
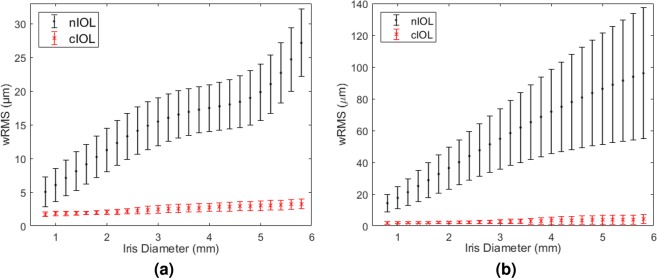
The average weighted root-mean-square spot-size (wRMS) ± standard deviation for centered intraocular lenses (IOLs) as a function of the iris diameter (**a**) in the normal group and (**b**) in the keratoconus group. The values of the aberration-neutral IOL (nIOL) are displayed as black dots, those of the custom IOL (cIOL) as red crosses.

An aberration-neutral IOL (nIOL) was chosen based on the wRMS for each eye. The anterior IOL surface was subsequently customized to correct the aberrations. The centered IOL with customized aberration-correcting front surface (cIOL) sucessfully reduced the wavefront-errors and the wRMS for normal cataractous eyes and even more for keratoconic eyes compared to the wavefront-errors and wRMS with the centered nIOL (Table 2, Fig. 1). This resulted in reduced dependency of the wRMS on the diameter of the virtual iris (Fig. 2). The improvement was particularly high in the keratoconus group. Typically, the differences between the IOL surface elevation of nIOL and cIOL were larger for keratoconic eyes than normal eyes. The standard deviation (SD) of the difference between the anterior IOL surface elevation of nIOL and cIOL was 8.4 μm for NP and 29.3 μm for KP.

**Table 2:** Weighted root-mean-square (RMS) spot-size and RMS wavefront-error (average ± standard deviation, range) with a centered intraocular lens (IOL) in the normal group and the keratoconus group.

Group	IOL	wRMS (μm)	RMS wavefront-error (μm)
normal	nIOL	25.53 ± 4.31 (16.4…30.3)	1.116 ± 0.268 (0.743…1.601)
	cIOL	2.40 ± 0.78 (1.5…4.5)	0.016 ± 0.004 (0.010…0.027)
keratoconus	nIOL	102.13 ± 37.8 (49.8…180.2)	5.540 ± 2.505 (2.325…11.66)
	cIOL	2.98 ± 1.87 (1.9…8.5)	0.017 ± 0.005 (0.012…0.026)

IOL: intraocular lens, cIOL: custom IOL, nIOL: aberration-neutral IOL, wRMS: weighted root-mean-square spot-size, RMS: root-mean-square.

### Impact of IOL Misalignment

The wRMS for random combinations of IOL- and iris-misalignment was analyzed with the Monte Carlo analysis method. The cIOL reduced the average wRMS compared to the nIOL for all eyes. The improvement was larger in the keratoconus group than in the normal group (Table 3). The average relative wRMS improvement with the cIOL compared to the nIOL given by $$100{\rm{ \% }}\times \langle wRMS({\rm{n}}{\rm{I}}{\rm{O}}{\rm{L}})-wRMS({\rm{c}}{\rm{I}}{\rm{O}}{\rm{L}})\rangle /$$$$\langle wRMS({\rm{nIOL}})\rangle $$ was 67.8% ± 6.1% (mean ± SD, range: 56% to 74%) for keratoconic eyes, and 36.5% ± 9.1% (range: 20% to 47%) for normal eyes. The differences between the minimum wRMS and the mean wRMS were largest with the cIOL indicating that misalignment had a larger influence on the wRMS with the cIOL compared to the nIOL. In 86.7% of all virtual implantations in the normal group, the cIOL reached a smaller wRMS than the nIOL. This was the case in 99.4% of the virtual implantations in the keratoconus group. The differences between wRMS with cIOL and nIOL were significant (*p* < 0.001) for all eyes.

**Table 3:** Average weighted root-mean-square spot-size (wRMS) and sensitivity characterized by the difference between minimal wRMS and mean wRMS (average over all patients ± standard deviation, range) in the normal group and the keratoconus group.

Group	IOL	wRMS (μm)	Sensitivity (μm)
normal	nIOL	16.4 ± 4.3 (8.1…34.6)	3.9 ± 1.7 (2.0…6.7)
	cIOL	10.3 ± 5.8 (2.0…47.3)	7.5 ± 1.8 (5.1…11.6)
keratoconus	nIOL	92.7 ± 34.4 (34.1…169.2)	11.7 ± 5.6 (6.2…23.6)
	cIOL	28.5 ± 18.6 (2.6…144.4)	24.7 ± 8.5 (12.6…238.0)

IOL: intraocular lens, cIOL: custom IOL, nIOL: aberration-neutral IOL, wRMS: weighted root-mean-square spot-size.

The correlations of the wRMS with the characteristic misalignment components were analyzed to determine which misalignment-components are most relevant. Iris decentration played a minor role for wRMS with the cIOL (significant correlation, *p* < 0.05, for only one keratoconic eye), but was significantly correlated to the wRMS with the nIOL in eleven normal and four keratoconic eyes. Unlike with the nIOL (significant correlation for two keratoconic eyes), the wRMS with the cIOL was significantly correlated with IOL decentration for all eyes (*p* < 0.001). The wRMS with the cIOL in eleven normal eyes and in five keratoconic eyes was significantly (*p* < 0.05) correlated with IOL tilt, while for the nIOL this was the case for eleven normal and two keratoconic eyes. IOL rotation was significantly (*p* < 0.05) correlated with wRMS in eleven normal and all keratoconic eyes with the cIOL. The axial position of the IOL had negligible influence on the wRMS (three times correlation with *p* < 0.05 for nIOL and cIOL), but was mostly responsible for the distance of the object point that was imaged onto the fovea.

**Figure 3:**
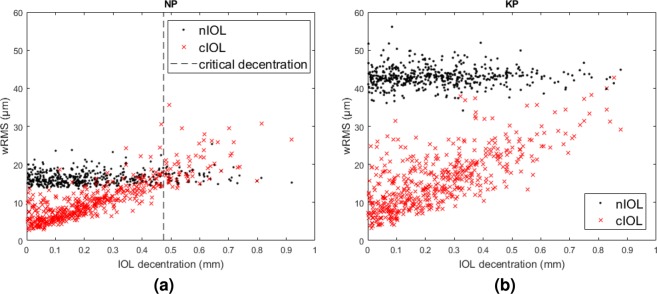
The weighted root-mean-square spot-size (wRMS) as a function of intraocular lens (IOL) decentration with an aberration-neutral IOL (nIOL, black dots) and a custom IOL (cIOL, red crosses) for (**a**) normal eye NP and (**b**) keratoconic eye KP. The critical decentration values were 0.47 ± 0.03 mm (NP, dashed line) and 1.19 ± 0.05 mm (KP).

IOL decentration appeared to be most relevant for the wRMS with the cIOL, but had little influence on the wRMS with the nIOL (Fig. 3). To give an estimate for the decentration-tolerance of the IOLs, we fitted the difference between the wRMSs with the cIOL and the nIOL with a linear function of IOL decentration and calculated its root. For normal patients, this critical decentration value was 0.51 ± 0.09 mm (mean ± SD, median: 0.54 mm, range: 0.35 mm…0.67 mm). For keratoconus patients, it was 1.29 ± 0.47 mm (median: 1.16 mm, range: 0.74 mm…2.24 mm). The fit had an adjusted *R*^2^ between 0.2 and 0.8.

## Discussion

A sufficiently precise mathematical surface representation of the cornael surfaces was achieved for all eyes, including eyes with advanced keratoconus. The surface representation was similar to the one used previously^18^. The weighted root-mean-square fit errors of the anterior cornea were slightly larger than the root-mean-square-errors reported there, because of the larger diameter of our fitted optical zone and the keratoconic corneas. The deviations of the surface representation from the data of keratoconic and normal eyes were below the precision of corneal surface measurements of the Pentacam HR for normal corneas^29^. This could not be achieved with *j* ≤ 28 Zernike polynomial terms^27^ for most keratoconic corneas, as expected. Even with 14 orders of Zernike polynomials, the anterior corneal surfaces of eyes with moderate to severe keratoconus could not be well fitted^17^. Fluctuations of corneal shape might exceed the precision of tomographic measurements^30^, and the precision might be worse for keratoconic eyes^31^. Ideally, the residuals of the fit to the corneal surfaces of keratoconic corneas should be compared to the shape-fluctuations for keratoconic corneas, but reliable data was not available. The precision of the fit with Zernike polynomials and fourth order basis-splines to the data points was sufficient.

Measurement uncertainty of the cornea propagates into the calculation of the cIOL, which is customized to correct the wavefront-errors. The uncertainty of the cIOL’s anterior surface ranges from ≈1 μm in the center to ≈5 μm in the peripheriy for normal patients, whereas about two third of this uncertainty are due to the anterior corneal surface shape. For keratoconic eyes, these values might be larger^31^. The use of the average values of multiple measurements should be recommended for custom IOL calculation to reduce the influence of statistical measurement uncertainties.

Wavefront-correction with a custom anterior IOL surface is as effective as with a posterior custom IOL surface: the spot-sizes and wave-front-errors for both examples (NP, KP) were smaller than in the examples provided by Zhu *et al*. with a custom posterior IOL surface^9^. The aim of the cIOL design was to correct all wave-front-errors. However, incomplete correction (e.g. with an IOL surface described by a reasonably low number of Zernike polynomial terms) may reduce the impact of misalignment on the optical properties of the pseudophakic eye^32^. The image quality with the centered cIOL was diffraction limited according to the Marechal criterion. The benefit from custom IOLs can be expected particularly large for eyes with abnormal corneal shapes and large pupils (especially under mesopic and scotopic conditions).

When implanted into a real eye, IOLs are often not perfectly aligned. To mimic this situation, the IOL and iris positions were varied in a simulated clinical study using Monte Carlo analysis. IOL decentration or IOL tilt may change the fixation axis and spherical equivalent refraction^33^. This was compensated by adapting the object point position, which might result in small changes of the magnification when comparing the wRMSs of different misalignment scenarios or between the IOL types. The difference between the object point distance with the cIOL and nIOL were 0.16 ± 0.69D in the keratoconus group and −0.16 ± 0.25D (mean ± SD) in the normal group.

Even in the presence of misalignment, the cIOLs produced smaller average wRMSs than the nIOLs. This is in agreement with previous studies concluding that aberration correction will result in improved image quality for moderate IOL misalignment^10,21,22,25,26^. Keratoconic eyes benefitted most from the cIOL. The average wRMSs with the cIOLs in keratoconic eyes were comparable to the average wRMSs of the nIOLs in normal eyes. The wRMS of both IOL types (cIOL, nIOL) in keratoconic eyes was affected more by IOL misalignment than in normal eyes.

The larger the corneal asymmetry, the larger the benefit that can be expected from customized IOLs compared to standard IOLs. In the normal group, the eye with the largest difference of the keratometric values (*K*_f_,*K*_s_) showed the largest reduction of the average wRMS with the cIOL compared to the nIOL, while the reduction was smallest for the eye with the smallest difference between the keratometric values. Parts of the reduction of the wRMSs (in normal and keratoconic eyes) were due to the correction of astigmatism, which could alternatively be corrected by spectacles or contact lenses. Correction with spectacles or contact lenses might reduce the difference in the performance of cIOLs and nIOLs as well as the impact of misalignment on the wRMS. Forthcoming studies including spectacle-simulation or comparing the performance of toric IOLs and cIOLs might lead to additional insights. In many normal corneas, the steep axis and the flat axis are not perpendicular to each other^34^, thus the aberrations can not fully be compensated with standard corrections (toric IOLs, spectacles, or contact lenses). Custom IOLs (such as the cIOL) might become an option for eyes with such an irregular astigmatism including keratoconic eyes with stable corneal tomography (e. g. after cross-linking) and eyes after corneal transplantation (penetrating keratoplasty, deep anterior lamellar keratoplasty).

Considering the different misalignment components, we found that IOL decentration is most critical for the wRMS with the aberration correcting cIOL. This is in agreement with previous studies^21,24,35^. We defined a critical decentration as the IOL decentration for which the average wRMS with the cIOL is expected to be equal to the average wRMS with the nIOL. To estimate the critical decentration a linear model was used. Deviations of the data from the model resulted in small adjusted *R*^2^. The deviations are mainly caused by the presence of the other misalignment compontens (iris decentration, IOL rotation, IOL tilt) and decentration having a different effect depending on its direction. We found average critical decentration of ≈0.5 mm for normal and ≈1.3 mm for keratoconic eyes. This is larger than the IOL decentration (0.30 mm for a 3 mm pupil and 0.38 mm for a 5.0 mm pupil) for which the image quality of an aberration-correcting IOL drops below that of an aberration-neutral IOL in a model eye with average spherical aberration according to Altmann *et al*.^26^. This can have four reasons: (1) We used the full corneal tomography of a small number of real eyes. These corneas not only provide spherical aberration, but other higher order aberrations as well. (2) We included further misalignment components aside from IOL decentration, which may partially compensate the effect of IOL decentration^24^. (3) We adjusted the position of the object point to correct the spherical refractive error^33^. (4) We used the wRMS to estimate the critical decentration, while Altmann *et al*. based their results on the modulation transfer function.

To keep complexity reasonable we restricted our study to monochromatic aberrations. Chromatic aberration could further increase the tolerance for IOL misalignment^22^. Average IOL decentration is ≈0.3 mm^21^. A large majority of patients could benefit from the wRMS reduction with the cIOL despite the effect of IOL decentration. Based on our Monte Carlo analysis, 86.7% of normal and 99.4% of keratoconic eyes will achieve a reduced (monochromatic) wRMS with custom IOLs compared to aberration-neutral nIOLs. Occasionally, larger IOL tilt (≥10°) and -decentration (≥1 mm) occur affecting about 10% of pseudophakic population^36^. In these cases, only eyes with highly asymmetric corneas are expected to benefit from cIOLs.

The wRMS with the nIOL showed significant correlation with iris decentration for most eyes. The iris position affects which part of the cornea is relevant for the image quality of the optical system. Tilt of the nIOL results in spherical and cylindrical refractive error^37,38^, which increase the wRMS with highly rotationally symmetric corneas, but can have a compensatory effect with astigmatic corneas. Tilt of the centered cIOL would always reduce the aberration-correction. IOL rotation around the *z*-axis significantly increased the wRMS of the cIOL in almost all eyes. The expected IOL rotation, for which the image quality becomes worse with wavefront-correcting IOLs than with the nIOLs (>28°)^39^, is larger than the rotations applied in our study.

The eye models used to optimize and test the IOLs have several limitations^33^. The most important are: fluctuations of the corneal shape, uncertainty in the placement of the virtual fovea, IOL and iris. Systematic measurement errors in assessing the (posterior) cornea might play a role for eyes with keratoconic corneal tomography. Changes of the cornea due to small incision cataract surgery^40^ were not included in the model. The long-term impact of surgically induced astigmatism is considered insignificant compared to random fluctuations of the corneal shape^30^.

Axial IOL placement used the prediction of the effective lens position by the Haigis formula^41^. The IOL constants were optimized according to the postoperative outcome with normal patients^42^. The axial positioning might be less reliable for keratoconic eyes. Ideally, the initial IOL alignment with respect to the videokeratometry axis should be based on average IOL alignment measured with the same IOL-model. Measurements with the nIOL^43^ became published recently, and were not available before our data analysis. The numerical ray tracing model also does not include the dependence of the IOL’s alignment from the phakic lens position^44^, since measurements of the phakic lenses were not available.

The results from the Monte Carlo analysis can only give clinical relevant predictions, if the distributions of the misalignment-components represent clinical observed data. Most studies report typical IOL tilt of 2…3° and IOL decentration of 0.2…0.3 mm, but larger values can be found in the literature as well^21,36^. Unfortunately, there were no estimations available for the specific nIOL. If tilt and decentration are modeled by independent Gaussian distributions, large IOL tilt combined with large IOL decentration becomes rare. We additionally varied the center of IOL tilt: If the IOL is not tilted around its center, IOL tilt will also decenter the IOL. The clinical relevance could additionally be improved by using an alternative image quality metric that is better correlated to visual performance than the wRMS^45^.

The monochromatic foveal wRMS significantly improved with the cIOL compared to the nIOL, even in the presence of IOL misalignment. The improvement was more pronounced for keratoconic eyes compared to normal eyes, as could be expected from an adaptive optics study^46^. In the same study, the authors also found that the visual performance after adaptive optics aberration-correction was significantly worse in keratoconic eyes compared to normal eyes. They argued that this might be due to the long term neural adaptation to a blurred image with keratoconic patients. However, they considered the visual performance a short time after aberration correction and neural adaptation to the improved image quality with cIOLs might take longer for keratoconic eyes.

A better understanding of the role of chromatic aberrations for human vision can help to improve simulations in the future. Polychromatic simulations require precise knowledge of the refractive indexes and their dependence on wavelength for all media involved. The method of Monte Carlo analysis could also be helpful to address the question which part of the aberrations should be corrected to allow substantial improvements of retinal image quality combined with reduced sensitivity for lens misalignment. These theoretical consideration could be done by comparing the results to the outcome with standard monofocal lenses (as presented here) or with toric lenses with or without additional spectacle corrections. Additional studies of the distribution and correlation between IOL tilt and IOL decentration will improve the parameters for future Monte Carlo analyzes.

In conclusion, monochromatic aberrations could be significantly reduced with custom IOLs, even in the presence of IOL misalignment. Larger benefits can be expected for eyes with abnormal corneal shape. We found that eyes with a stable keratoconic tomography pattern could benefit more from custom IOL optic-design than normal eyes despite a larger impact of IOL misalignment on the foveal spot-size.

## Methods

### Measurements

The measurements included corneal tomography with Pentacam HR (Oculus Optikgeräte, Wetzlar, Germany) and ocular biometry with IOLMaster 500 or IOLMaster 700 (Carl Zeiss Meditec, Jena, Germany). The anonymized data of normal eyes belong to patients who underwent cataract surgery at the Rudolf-Virchow eye-hospital in Glauchau (Germany). The data from keratoconic eyes were extracted from the database of the Homburg keratoconus center (HKC) at Saarland University Medical Center (UKS) in Homburg/Saar (Germany)^47^. The data was analyzed retrospectively. The analysis was approved by the institutional review board of Saarland University, and conducted in accordance with the guidelines of the declaration of Helsinki. All patients provided informed consent to the use of their data. Only patient’s without any other eye disease other than cataract or refractive error were included. Patients in the keratoconus group were required to have a TKC-index^48^ ≥1.

### Corneal Representation

In clinical practice, IOL calculation is usually performed based on keratometry readings. To include higher order aberrations in the IOL calculation and simulation of the pseudophakic optics, we used the individual corneal tomographies, measured with the Pentacam HR. To enable ray tracing through the corneal tomography measurements, the elevation height data of anterior and posterior cornea were exported and fitted to a mathematical surface model: The exported height data was approximated with 11 Zernike polynomials (*j* ≤ 11^27^) plus fourth order basis-splines^28^ within the central 10 mm of the cornea. The basis-splines were set up with regular support in *x*- and *y*- direction (*dx* = *dy* = 0.5 mm) to facilitate the numerical ray tracing. Weighted least squares approximation to the corneal elevation data was used to calculate the Zernike polynomial coefficients and fit the residuals with basis-splines. A smoothing term reducing the second derivatives of the surface height at the data points was included in the fitting of the basis-splines. The weights were based on the precision estimated for the corneal shape measurement with Pentacam HR for normal subjects^29^.

### IOL Optimization

Custom IOLs were calculated and analyzed using personalized numerical ray tracing eye models. The models were built in the same way as those presented in a previous study^33^, but had a modified corneal representation and virtual iris. Instead of the Zernike polynomial representation of the cornea, the mathematical description using a combination of Zernike polynomials and basis-splines was used. To calculate the IOL, the virtual iris was replaced by weighting each ray at the IOL’s anterior surface. The weights were ≡1.0 in the inner 3 mm of the IOL and decreased linearly with the radius to zero at the IOL’s edge (diameter: 6 mm). They were applied in addition to the weights at the corneal plane simulating the Stiles-Crawford effect.

We used two types of IOL: the aberration-neutral nIOL, and the fully aberration correcting cIOL. The nIOL was the Aspira-aA (HumanOptics AG, Erlangen, Germany), whose design data was provided by the manufacturer. The IOL power which produced the smallest wRMS was chosen in the same way as described previously^33^. The Cartesian *x*, *y* coordinates of the object point *z* = −6 m in front of the corneal apex were adjusted so that it produced the minimum wRMS at the position of the virtual fovea. The back surface of the IOL was kept and the front surface customized to calculate the cIOL.

During customization, the IOL’s anterior surface was modified to nullify the optical path length differences in an iterative manner, similarly as described previously^9^ for customization of the IOL’s posterior surface. First, the ideal wavefront inside the IOL was calculated using backward numerical ray tracing from the virtual fovea through the IOL’s posterior surface. The wavefront can easily be propagated inside the IOL, because the rays travel perpendicular to the wavefront. Using forward numerical ray tracing starting from the object point, the intersection of each ray with the propagated wavefronts were computed and the corresponding propagation lengths were iteratively adjusted to minimize the optical path length differences. The coordinates of the IOL’s ideal anterior surface were given by the intersection of the rays with the wavefronts propagated by the respective propagation lengths through the IOL. The cIOL’s minimum thickness was imposed to be 5 to 8 µm thicker than the edge thickness of the nIOL to ensure mechanical stability. The coordinates of the anterior IOL surfaces were fitted with a smooth mathematical surface description (comination of Zernike polynomial terms with *j* ≤ 22^27^ and fourth order basis splines with *dx* = *dy* = 0.375 mm knot distance) to enable numerical ray tracing. The precision of the fit was in the sub μm scale and sufficient to achieve root-mean-square wave-front errors of <0.0027 μm for all eyes.

The wRMS and the wavefront-error were used to verify successful optimization. Backward numerical ray tracing from the virtual fovea through IOL and cornea was used to calculate the wavefront in front of the eye, and the best-fitting sphere was subtracted from the wavefront to calculate the wavefront-errors. To analyze the dependency of wRMS on the iris diameter, the wRMSs were calculated without weighting the rays at the IOLs’ surfaces. The iris diameter was simulated as centered aperture stop placed at the anterior chamber depth.

### IOL Misalignment

Monte Carlo analysis was used to study the effect of misalignment on the wRMS with both IOL types in a similar way as described previously^10,25,26^. When analyzing the IOL’s optical properties inside the numerical ray tracing models, a round virtual iris aperture at the preoperative anterior chamber depth replaced the weighting of the rays at the IOLs’ surfaces. The iris diameter of 4.5 mm corresponds to the larger diameter in front of the IOL, that is recommended for testing its optical properties^49^. A total of 500 virtual implantations were simulated for each eye and IOL type. The misalignment of the nIOL and the cIOL were identical to enhance comparability. IOL and iris were randomly misaligned. The (pseudo-random) magnitude of misalignment was drawn from Gaussian distributions with the following width (*σ*):

- Lateral IOL decentration relative to the videokeratometry axis (Δ*r*): 0.3 mm^21^,
- Axial IOL displacement (Δ*z*): 0.3 mm^50,51^,
- IOL tilt (rotation around an axis in the *x*/*y*-plane: ${\rm{\Delta }}{\alpha }_{x,y}=\sqrt{{\rm{\Delta }}{\alpha }_{x}^{2}+{\rm{\Delta }}{\alpha }_{y}^{2}}$): 2.6°^21^,
- IOL rotation around the *z*-axis (Δ*α*_*z*_): 7°^52,53^,
- Axial distance between the center of the IOL and the center of IOL tilt (Δ*Rot*): 2.0 mm,
- Iris decentration relative to the videokeratometry axis (Δ*r*_iris_): 0.2 mm^10^.

The direction of each misalignment-component was defined based on uniform pseudo-random numbers. All pseudo-random numbers were generated using the Mersenne-Twister-Algorithm^54^ whose seeds were setup in a way that avoids significant (*p* < 0.05) correlations between the misalignment-components. The object point was adjusted to calculate the wRMSs on the virtual fovea^33^.

The wRMSs with the different IOL models were compared using the Wilcoxon-test for paired data. Correlations (Pearson) between characteristic misalignment components (IOL decentration |Δ*r*|, IOL tilt |Δ*α*_*x*,*y*_|, IOL rotation |Δ*α*_*z*_|, axial IOL displacement |*z*| and lateral iris decentration |Δ*r*_iris_|) and the wRMS were analyzed. The significance level was set to *p* = 0.05.

## Supplementary information

Dataset 1

## Data Availability

The datasets generated during the current study are included in the Supplementary Information files: **S1: Keratoconus.xlsx** contains the results of the Monte Carlo analysis for keratoconic eyes. **S2: Normal.xlsx** contains the results of the Monte Carlo analysis for normal eyes. **S3: KeratoconusPatients.xlsx** contains the biometric data of the keratoconic eyes. **S4: NormalPatients.xlsx** contains the biometric data of the normal eyes.
